# Determination of Loline Alkaloids and Mycelial Biomass in Endophyte-Infected *Schedonorus pratensis* by Near-Infrared Spectroscopy and Chemometrics

**DOI:** 10.3390/microorganisms8050776

**Published:** 2020-05-21

**Authors:** Giovanni Cagnano, Beatriz R. Vázquez-de-Aldana, Torben Asp, Niels Roulund, Christian S. Jensen, Milton Carlos Soto-Barajas

**Affiliations:** 1 DLF Seeds A/S, Højerupvej 31, 4660 Store Heddinge, Denmark; gca@dlf.com (G.C.); nr@dlf.dk (N.R.); csj@dlf.com (C.S.J.); 2 Department of Molecular Biology and Genetics, Faculty of Science and Technology, Aarhus University, 8000 Aarhus C, Denmark; torben.asp@mbg.au.dk; 3 Institute of Natural Resources and Agrobiology of Salamanca (IRNASA-CSIC), 37008 Salamanca, Spain; beatriz.dealdana@irnasa.csic.es; 4 Cátedras-CONACYT, Instituto Tecnológico de Chiná, 24520 Campeche, Mexico

**Keywords:** *Epichloë*, fungal endophytes, meadow fescue, NIRS, chemometrics

## Abstract

Near infrared spectroscopy (NIRS) is an accurate, fast and nondestructive technique whose use in predicting forage quality has become increasingly relevant in recent decades. *Epichloë*-infected grass varieties are commonly used in areas with high pest pressure due to their better performances compared to endophyte-free varieties. The insect resistance of *Epichloë*-infected grasses has been associated with four main groups of endophyte secondary metabolites: ergot alkaloids, indole-diterpenes, lolines and peramine. Concentrations of these alkaloids are usually measured with high performance liquid chromatography or gas chromatography analysis, which are accurate methods but relatively expensive and laborious. In this paper, we developed a rapid method based on NIRS to detect and quantify loline alkaloids in wild accessions of *Schedonorus pratensis* infected with the fungal endophyte *Epichloë uncinata*. The quantitative NIR equations obtained by modified partial least squares algorithm had coefficients of correlation of 0.90, 0.78, 0.85, 0.90 for *N*-acetylloline, *N*-acetylnorloline and *N*-formylloline and the sum of the three, respectively. The acquired NIR spectra were also used for developing an equation to predict in planta fungal biomass with a coefficient of correlation of 0.75. These results showed that the use of NIRS and chemometrics allows the quantification of loline alkaloids and mycelial biomass in a heterogeneous set of endophyte-infected meadow fescue samples.

## 1. Introduction

The physical, non-destructive technique of near-infrared spectroscopy (NIRS) has dramatically changed the analysis of very different materials, especially in agricultural and food industries [1]. Using NIRS it is possible to predict some traits of interest in materials due to the light absorption by the surface of a sample using incident polychromatic light over a spectral wavelength in the infrared region (from 1100 to 2000 nm). The sample absorbs and reflects specific frequencies depending on the chemical bonds within the constituent of the matrix [2], thus allowing the identification of specific regions of the spectrum associated with protein, fiber, starch, alkaloid, and other compounds.

NIRS is not a ‘standalone’ technique; it requires calibration with standard reference samples previously analyzed by conventional wet chemistry in order to find an adequate correlation between the NIR-spectra and the chemical composition of the sample. Therefore the validity of the NIRS calibration equation obtained will never be better than the databases used to establish the calibrations [3]. The statistical analysis on which calibrations are based, called “chemometrics”, include multiple linear regression, Fourier transform, full spectrum, and non-linear models [4] with the aim to obtain a prediction equation for quantification of one or more constituents from a sample. From the 1960s, when the technique was first described [5], its use has extended widely thanks to the widespread use of monochromators, to the development of user-friendly software for chemometrics, to the versatility of this method and to the high amount of information that is possible to infer from a single NIR analysis. Almost ten years later NIRS was first used to measure forage quality [6] and since then became increasingly relevant to the extent that now it is a routine analysis technique in forage research and breeding. Most of these analyses predict aspects about forage composition in terms of chemical fractions such as crude protein, neutral-detergent fiber, acid-detergent fiber, water-soluble carbohydrates, lignin and dry-matter digestibility [6,7,8] and recently other parameters such as alkaloids [9]. Profiling forage grass cultivars at all these traits gives breeders valuable information on which they can base their selection for elite varieties with a high digestibility [10] or particular characteristics such as alkaloid profile [9,11].

Relevant forage and turf species such as perennial ryegrass (*Lolium perenne*), tall fescue (*Schedonorus arundinaceus*), meadow fescue (*Schedonorus pratensis*) and red fescue (*Festuca rubra*) form symbiotic relationships with filamentous fungi of the Clavicipitaceae family belonging to the genus *Epichloë* [12]. When an endophyte-infected seed germinates, the fungus grows systemically throughout the aboveground tissue; and in strictly asexual strains, the endophyte reaches the inflorescences where it initially colonizes the ovule, then the embryo and endosperm of the developing seeds that will form the next grass generation [13].

In the last 20 years *Epichloë*-infected cultivars (E+) of perennial ryegrass and tall fescue became crucial in New Zealand, Australia and USA due to their enhanced performances compared to endophyte-free (E−) varieties in environments with strong selective pressure [14,15]. Insect resistance of E+ varieties has been associated with an array of secondary metabolites produced by *Epichloë* endophytes that are usually grouped in four classes: ergot alkaloids, indole-diterpenes, lolines and peramine [16,17]. Identification and quantification of these alkaloids are crucial because ergot alkaloids and indole-diterpenes have been identified as the cause of fescue toxicosis and ryegrass stagger, respectively [18,19]. Peramine and lolines, on the other hand, are livestock friendly and have insecticidal and insect feeding deterrent activities [20,21,22].

Nowadays, quantitative analysis of alkaloids is mostly based on high performance liquid chromatography (HPLC) and on gas chromatography [23,24,25]. These methods allow high precision measurements, but they use relatively complicated protocols, expensive chemicals and instruments. As an alternative technique for detecting alkaloid concentration, NIRS has been successfully used to quantify ergot alkaloids (in tall fescue and perennial ryegrass), indole diterpenoids and peramine (in perennial ryegrass) [9,26] but not for lolines. Lolines derive from homoserine and proline and are classified as aminopyrrolozidine. According to the modification of the 1-amino group they can be found in different forms, the most common and abundant are *N*-acetylloline (NAL), *N*-acetylnorloline (NANL) and *N*-formylloline (NFL) [27,28,29]. Among grass species of agricultural interest, lolines can be found in tall fescue plants infected with *E. coenophiala* or in meadow fescue plants infected with *E. uncinata* or *E. siegelii.*

Loline alkaloids are positively correlated with fungal biomass, the more fungal cells colonize the host, the higher levels of alkaloids are produced [30]. Thus, in planta mycelial biomass should also be considered a trait of interest when aiming to achieve high concentrations of lolines in order to ensure a wide spectra resistance against insects. Several methods have been described to quantify the endophyte concentration in host tissues: by counting endophyte hyphae previously stained with aniline blue in a leaf cross-sections [31]; by using quantitative PCR (qPCR) [32,33,34]; by using an ELISA-based protocol [35]. Despite a variety of strategies, quantification of *Epichloë* mycelium in experimentation or practice in the context of forage companies did not spread.

The aim of this work was to evaluate the suitability of NIR spectroscopy for quantitative analysis of loline alkaloids and mycelial biomass in meadow fescue plants infected with the fungal endophyte *E. uncinata*.

## 2. Materials and Methods

### 2.1. Plant Material

The samples for this study were a selection of 216 meadow fescue plants infected with *Epichloë uncinata* previously characterized by Cagnano et al. [36]. This dataset included 135 plants collected in wild and semi-wild meadows in different countries in Europe, 65 plants from accessions requested to The Nordic Genetic Resource Centre (NordGen) and 19 plants from accessions requested to the United States Department of Agriculture (USDA). Endophyte-free plants were collected in the same populations and they were used as a negative control of alkaloid production. Infection status of all the samples was determined using the immunoblot assay “Phytoscreen Field Tiller Endophyte Detection Kit” (Cat. #ENDO797-3; Agrinostics Ltd. Co., Watkinsville, GA, USA) described by Hiatt et al. [37] following manufacturer’s description. In order to reduce variability due to different growth stages, plants were trimmed very short (approximately 5 cm), cloned in pots (35 × 30 cm) and left growing in a greenhouse, with 16 light hours at 15–24 °C, for three months. Samples from the basal part of the tiller were harvested and immediately freeze-dried. Samples were subsequently powdered in a laboratory mill with a grind size of approximately 5 μm.

### 2.2. Reference Analysis of Loline Alkaloids and Mycelial Biomass

**Figure 1:**
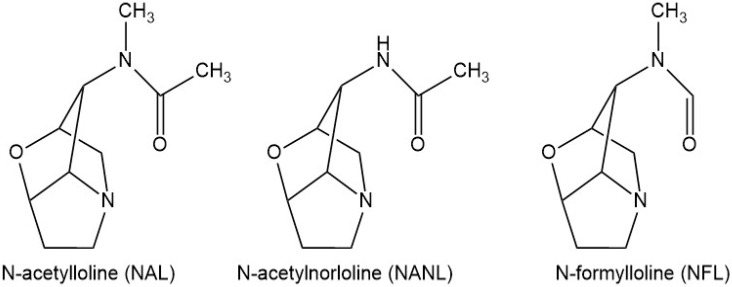
Chemical structures of *N*-acetylloline (NAL), *N*-acetylnorloline (NANL), *N*-formylloline (NFL).

About 50 mg of each sample was analyzed in duplicate in AgResearch Grasslands (Palmerston North, New Zealand). Loline alkaloids *N*-formylloline (NFL), *N*-acetylloline (NAL), *N*-acetylnorloline (NANL) (Figure 1) were measured in all 216 samples using a gas chromatographic (GC) method described by Baldauf et al. [26]. The limit of detection of this method, for loline alkaloids, was 10 ppm, while limit of quantitation was 25 ppm.

A subset of 100 plant samples, selected to include both accessions (NordGen and USDA) and collected plants with different lolines concentrations (min = 26 mg∙kg^−1^; max = 3435 mg∙kg^−1^; mean = 1553 mg∙kg^−1^; SD = 1064 mg∙kg^−1^), was used to measure the mycelial mass using an ELISA-based analysis as previously described in Cagnano et al. [38] following Faville et al. [35].

### 2.3. Acquisition of Infrared Spectra

**Figure 2:**
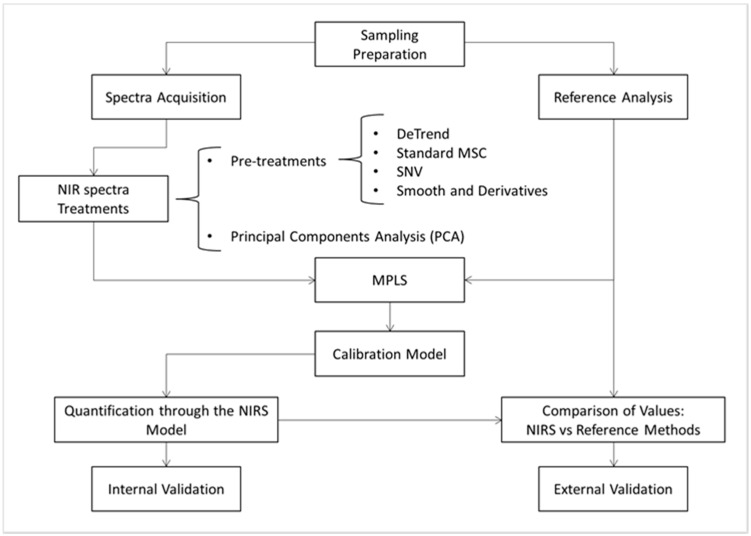
Schematic representation of the steps followed for quantitative analysis in near infrared spectroscopy.

Figure 2 summarizes the schematic process for the development of NIRS equations for quantification of lolines alkaloids and mycelial biomass.

The first step in NIRS is acquisition of the plant sample spectra; this stage was performed at the Department of Analytical Chemistry, Nutrition and Bromatology, University of Salamanca (Spain). For recording the NIR spectra a 1.5-m fiber optic probe with a remote reflectance system, of the 210/7210 Bundle (beam) regular type, with a 5 × 5 cm quartz window was applied directly on 2.0 g of each freeze-dried ground sample of meadow fescue. Spectra were stored in a NIR System Foss5000 spectrometer (FOSS Analytical, Hillerød, Denmark). Instrument control, manipulation of spectral files and chemometric analyses were made with the software WinISI 1.50 (International, LLC, State College, PA, USA).

Each of the recorded spectra was measured at intervals of 2 nm within the range from 1100 to 2000 nm. The resulting record was the average of 32 readings both in the grass sample and a ceramic plate used as reference material. To minimize sampling error all ground grass samples were analyzed in triplicate without moving the fiberoptic probe. The mean of these replicates was used in the statistical analysis, stored as the reciprocal logarithm of reflectance (log 1/R, R = intensity of reflected light at each wavelength) and used for further chemometrical analyses.

In order to identify and select the mathematical treatments more effective to reduce the signal/noise ratio from spectra and stabilization of the baseline, we evaluated the effect of reduction of gaps and application of derivatives and smoothing, in combination with four transformations: averaging, standard normal variate (SNV), de trending (DT), and a combination between SNV and DT [39,40]. A notation of four digits (a, b, c, d) was assigned to identify each transformation of the spectra: derivative order (a); derivative applied points (b); points on the first smoothing (c); points on the second smoothing (d) [41].

The collected spectra were divided randomly using an automatic algorithm from the WinIsi software into two subsets, one of them (ca. 75% of all the samples) was used for calibration of the NIRS models, and the rest of samples (ca. 25%) were separated to corroborate, by external validation, the performance of the obtained NIR equations. The characteristics of meadow fescue samples are indicated in Table 1.

### 2.4. Training and Calibration of the NIRS Models

Spectral data from the calibration set were analyzed by a principal component analysis (PCA) generating 20 different files by the combination of the mathematical treatments (spectra averaging, SNV, DT, SNV + DT, smoothing, gaps and derivatives) as described above. Anomalous spectra were identified by using the Mahalanobis distance (H-statistic, samples with *H* > 3.0) and discarded.

A modified partial least squares (MPLS) regression method was used to obtain the NIRS equation for alkaloids and mycelial biomass. MPLS is similar to partial least squares (PLS) regression, but more stable and accurate. Similar to principal component regression, the PLS is based on a reduction of variables but the calibration process uses both the reference data and spectral information to form the factors useful for the fitting purposes [42]. The modification in MPLS consists of a standardization of the NIRS residuals at each wavelength, after one factor is calculated the residual is divided by the standard deviations before calculating the next factor. MPLS equations also were optimized by using mathematical treatments (MCS; SNV; DT and SNV-DT) to avoid spectra scattering effects. MPLS was applied on the 20 files generated by the PCA, creating a combination of other 20 pre-treatments, and obtaining a total of 400 different equations to be evaluated for the quantification of each alkaloids and the mycelial biomass.

A cross-validation process was applied to select the optimal number of factors and to avoid overfitting [41]. Cross-validation is a subsequent series of comparisons between groups; in each step one group is alternatively evaluated using candidate equations developed from the remaining set. Differences among actual and calculated concentrations in each comparison are combined to determine the standard error of cross-validation (SECV). The SECV indicates the overall accuracy of the equation and it is taken to define the number of factors required in the evaluated equation. A removal process of atypical samples is applied twice during cross-validation, outliers are identified through the *T* value (residuals/SECV) and all samples that surpass a *T* value higher than 2.5 are eliminated from the calibration set.

The selection of the best NIRS equations for alkaloid quantification was based on the following statistical methods: (i) the multiple correlation coefficient (RSQ) which measures the fitting degree between predicted data and actual concentration; (ii) the Standard Error of Calibration (SEC), an estimate of the best accuracy obtainable using the specific wavelengths of the calibration equation; (iii) Residuals, differences between the actual value $y_{i}$ and the predicted value; (iv) BIAS, the medium value of the residuals; (v) the Standard Error of Cross Validation (SECV), and (vi) the ratio of standard deviation (SD) to SECV of the data set, known as RPD ratio which according to Williams and Sobering [43] is desired to be larger than 2.0 for good calibration.

Robustness of the NIR models for alkaloids and mycelial biomass quantification were corroborated through external validations with samples not included in the calibration set (Table 1). The NIRS-predicted and the reference data (GC, or mycelial biomass) were compared through a Student’s *t*-test for paired values, and the residuals were also calculated.

## 3. Results

### 3.1. Chemical Measurement

Concentrations of NAL, NANL and NFL were measured in 216 samples of endophyte-infected *S. pratensis*. Non-infected plants did not produce the alkaloids. Results of the chemical measurement have been described in Cagnano et al. [36]. Briefly, there was a wide variation of the total concentration of lolines, calculated as sum of the concentrations of the single compounds (NAL, NANL and NFL), spanning from barely detectable traces (<25 mg∙kg^−1^) up to 5629 mg∙kg^−1^ (Table 1).

NFL was the most abundant loline alkaloid accounting, on average, for 73% of total lolines, followed by NANL and NAL accounting for 16% and 11% of total lolines, respectively. Differences in the proportion of the three alkaloids were found at increasing concentrations, suggesting a trend according to which the production of high levels of lolines is correlated with a slight but significant increase in the proportion of NANL and NFL (R^2^ < 0.10) and with a greater decrease in NAL (R^2^ < 0.54).

Fungal concentration was measured in a subset of 100 samples used for lolines quantification. Values ranged from 0.22 to 3.97 mg∙g^−1^ with an average of 1.45 mg∙g^−1^ (95% CI ± 0.19; SE = 0.1; *n* = 100). In Cagnano et al. [38] a significant positive correlation between concentrations of loline alkaloids and in planta fungal biomass was measured using the non-parametric Kendall rank correlation coefficient (τ = 0.48; *p* = 2.5 × 10^−10^).

### 3.2. NIR Analysis

**Figure 3:**
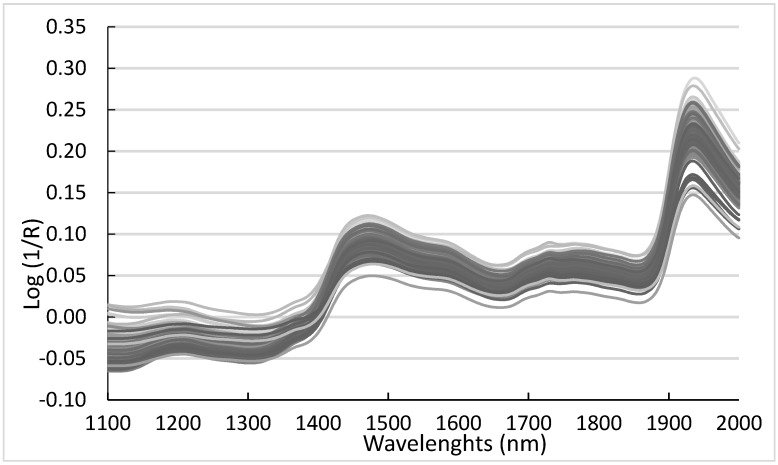
Spectra from NIR range (1100–2000 nm) of the 216 samples of endophyte-infected *Schedonorus pratensis*.

Figure 3 shows the NIR spectra of the 216 E+ meadow fescue samples used in this study within the range of 1100–2000 nm.

Table 2 presents the results obtained after PCA was applied on the spectra with the mathematical treatments, numbers of principal components, variability explained, and spectral outliers. The variability explained by each model ranged from 98.88 to 100% and the number of outliers detected was relatively low compared to the total number of analyzed samples.

The development of the quantitative models was done through the modified partial least squares method (MPLS) using the spectra and the reference concentrations of each alkaloid (NAL, NANL, NFL), and for mycelium biomass. In this procedure samples in which the alkaloid concentration was zero in the chemical analysis (GC) were not included.

For each alkaloid and for the mycelium biomass a calibration model was developed as follows. There are two types of outliers, spectral outliers (H > 3) which means that they are too different than the average signal of the group, and the chemical outliers (T > 2.5) whose residuals are bigger enough therefore the laboratory and the NIRS results are not similar and they cannot be compared so including outliers in the development in the NIRS equations will affect they performance. After PCA, the number of PCs was selected and the spectral outliers (H > 3) were eliminated; then the mathematical treatments were applied, as explained in the Material and Methods section having 400 equations to be evaluated for every loline alkaloid and the mycelia mass. The quantitative equation with the best statistical parameters (RSQ, SEC, SECV, and RPD) was selected and a cross validation was performed. Chemical outliers (T > 2.5) were eliminated for optimization of the equations, then the errors of prediction (SEP and SEPc) and prediction ability (RPD) were calculated.

After analyzing samples identified as outliers, reported in Table 2, no particular pattern or cluster was detected; on the contrary, they were from different origins and had high heterogeneity in their characteristics. Moreover, as NIRS is affected by many causes such as humidity, plant variety, phenological state of the plants, and fungus species, the reasons for why those samples were identified as outliers are indefinite.

### 3.3. Quantification of N-Acetylloline (NAL)

The best results of calibration for NAL quantification by NIR were obtained using the spectral pre-treatment n3 (no scattering), with the numerals (2,10,10,1). Thirteen principal components were required to explain 99.75% of the spectral variability among samples in the calibration set. Six samples were eliminated by the H criterion (Table 3).

The MPLS best performance for NAL quantification was obtained with the pre-treatment s2 and using ten PLS factors. The final calibration set was constituted by 143 samples because six samples were eliminated using the T criterion. The NIR model had an RSQ of 0.778, the lowest among the measured alkaloids, with SEC and SECV of 32 and 51 mg∙kg^−1^, respectively (Table 3).

**Figure 4:**
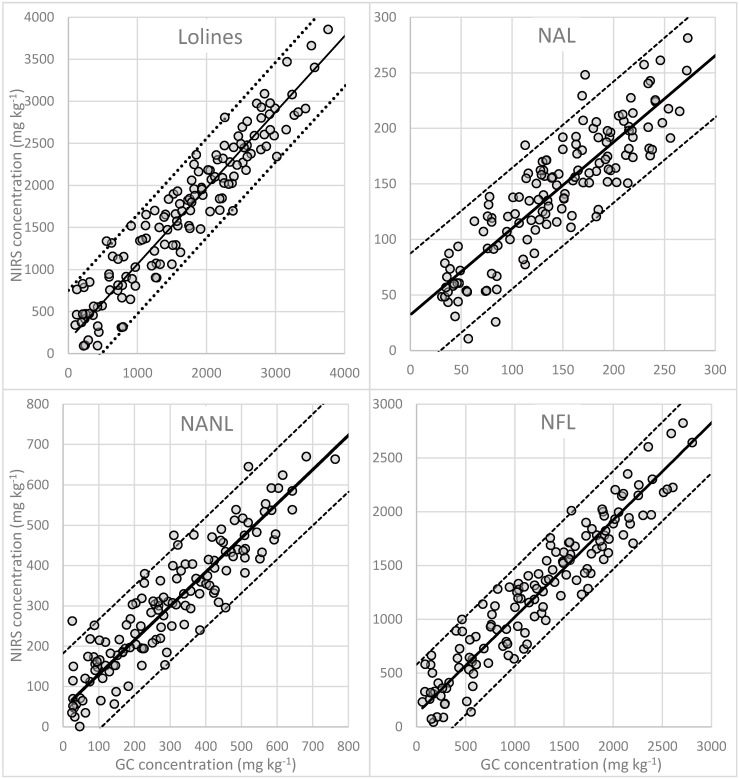
Internal validation comparing the concentrations of *N*-acetylloline (NAL), *N*-acetylnorloline (NANL), *N*-formylloline (NFL) and total lolines (as the sum of three alkaloids) obtained with conventional methods (GC) and the ones predicted by NIR spectroscopy using the modified partial least squares (MPLS) regression. The IC 95% is represented by the dashed lines.

Internal validation of the model, by comparing the concentration of NAL obtained with GC with the values using NIRS equation (Figure 4) allowed the calculation of the SEP which was 31 mg∙kg^−1^ and the predictive capability of the NIRS equation (RPD = 2.129). External validation of the NIR equation for quantification of NAL and the GS concentrations showed a *p*-value of the t-test of 0.02, therefore, the results of prediction are acceptable. 

### 3.4. Quantification of N-Acetylnorloline (NANL)

The model with the best performance for NANL quantification by NIRS was obtained when spectra were transformed by the mathematical treatment s2: standard normal variate (SNV) with the numerals (2,4,4,1) in the PCA, with twenty-seven factors that explained 98.91% of the spectral variability. In this process, four spectral outliers were detected and eliminated (Table 3).

In the MPLS regression the mathematical treatment used was m2 which consists of the application of SNV + DT transformations correction of trend with the numerals (2,4,4,1) and ten PLS factors. The model for quantification of NANL had an RSQ of 0.836, a standard error of calibration of 77 mg∙kg^−1^ and the standard error of cross-validation was 141 mg∙kg^−1^ (Table 3).

Comparing actual NANL concentration with those values predicted by NIRS, it was found that the SEP was 74 mg∙kg^−1^ and the RPD was 2.56. Taking into account this RPD value, it is possible to use the NIRS models for quantification of NANL in meadow fescue samples (Figure 4). Results of the external validation, in which the performance of the NIR equation for quantification of NANL was evaluated, indicated that the concentrations calculated were on the edge of the chosen statistical significance (*p* = 0.048) therefore, as for NAL, NIRS technology showed a good prediction capacity for the quantification of NANL. The RMSE in the calculation of the concentration using NIR was 184 mg∙kg^−1^ and the residuals 151 mg∙kg^−1^.

### 3.5. Quantification of N-Formylloline (NFL)

The model with the best results for NFL was obtained using the spectral pre-treatment n0 (no scattering), with the numerals (0,0,1,1). Nine principal components were required to explain 99.99% of the spectral variability and four samples were eliminated by the H criterion.

Similarly to NANL, the mathematical treatment that showed the best results in the MPLS regression was the m^2^ using ten PLS factors. The final calibration set was obtained with 146 samples because seven samples were eliminated using the T criterion. The calculated RSQ of this NIRs model was 0.901, the highest among the measured loline alkaloids, with a SEC and a SECV of 251 and 519 mg∙kg^−1^, respectively (Table 3).

The correlation between the concentration of NFL obtained with GC with the values using NIRS was 0.901 (Figure 4). The SEP was 240 mg∙kg^−1^ and the predictive capability of the NIRS equation was 3.195. These statistical parameters show that the NIRS model for quantification of NFL works better than the other two loline alkaloids: the predicted concentrations were reliable and not significantly different from results obtained with GC (*p* = 0.265).

### 3.6. Quantification of Total Lolines

The model selected for the quantification of the total amount of loline alkaloids (defined as the sum of the concentrations of NAL, NANL and NFL), was obtained when the sample spectra were transformed using the mathematical pre-treatment n0: no scattering (0,0,1,1). Nine factors were required for PLS regression and explained 99.99% of the spectral variability (Table 3). As is shown in Table 3 for total lolines, we obtained a model developed using 146 samples, and only four spectral outliers were detected and then eliminated according to the *H* criterion. On the other hand by using the *T* criterion (high residual, *T* > 2.5) seven chemical outliers were identified. This equation, designed for quantification of total lolines, had a RSQ = 0.897 which is indicative of a high correlation between the conventional and NIRS methods; a standard error of calibration (SEC) of 324 mg∙kg^−1^ and a standard error of cross-validation (SECV) of 667 mg∙kg^−1^ (Table 3).

The uncertainty in the prediction due to the model is indicated by the standard error of prediction (SEP), the standard error of prediction corrected (SEPc) and by the residual (BIAS) obtained by means of internal validation. The correlation between the reference values and the ones predicted by NIRS samples from the calibration set is presented in Figure 4. The predictive capability of the model (RPD) was 3.252 indicating that the obtained model can be applied to estimate accurately total lolines concentration in meadow fescue samples.

### 3.7. Quantification of Fungal Mycelium in Planta

As regards the quantification of fungal mycelium in planta, the spectral pre-treatment s0 (SNV) with the numerals (0,0,1,1) was the most accurate model. Four principal components explained 98.91% of the spectral variability and four spectral outliers were detected. Calibration was based on 65 samples and two samples were eliminated according to the T criterion. The mathematical treatment d2, using 10 PLS factors, was the one that gave the best results in the MPLS regression (Table 3).

**Figure 5:**
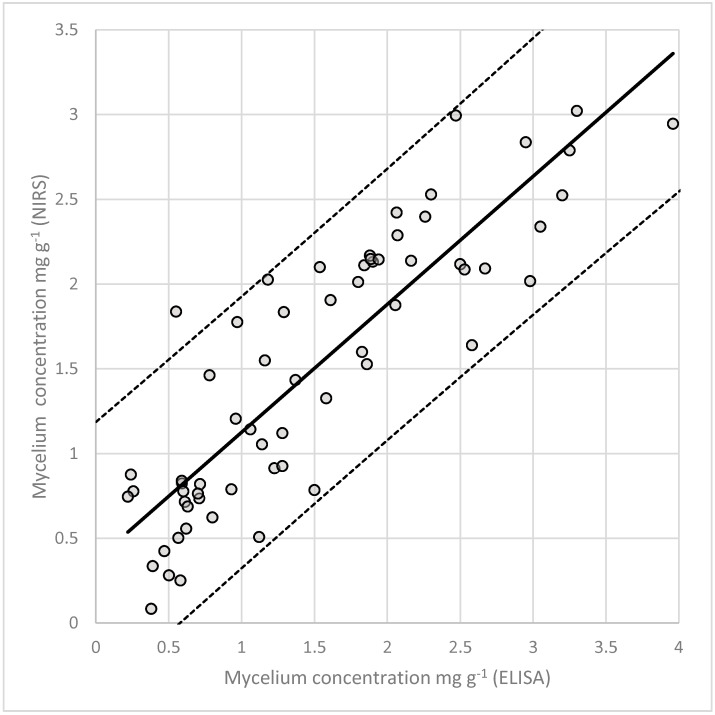
Internal validation comparing the concentrations of in planta fungal mycelium measured with an ELISA test and the ones predicted by NIR spectroscopy using the MPLS regression. The IC 95% is represented by the dashed lines.

The calibration equation had a calculated RSQ of 0.754 (Figure 5), lower than the loline alkaloids, accordingly its predictive capability was 2.036. This was expected considering the lower number of samples. These data suggest that the prediction of fungal biomass using this model can be difficult and results should be taken cautiously but the equation could be improved by adding samples to the calibration set.

## 4. Discussion

The objective of this study was to investigate the suitability of NIRS to quantify loline alkaloids and fungal biomass in *E. uncinata*-infected meadow fescues. The results show that the spectral information obtained directly from powdered meadow fescue plant samples can be used to reliably quantify the total amount of lolines and NFL. The quantification of these alkaloids was accurate and highly correlated with data obtained from conventional methods (GC). The prediction models to estimate the concentrations of NANL, NAL and fungal mycelium were less precise but still able to give an estimation of their in planta concentrations. This report is the latest in a series of studies about the use of NIRS to quantify in planta grass endophyte alkaloids [9,27]. This technique can be a helpful and relatively cheap tool in studies with a high number of samples or in breeding programs of companies working with E+ grass varieties where NIRS is already widely used to phenotype grass varieties.

The NIRS equation for quantification of the total amount of loline alkaloids had high accuracy (RSQ > 0.90) despite the fact that the set of samples was composed of wild meadow fescue accessions with diverse origins, conditions and alkaloids concentrations, indicating the high robustness of this method. If this method was applied to a single cultivar, the accuracy could have been increased even further because of the higher homogeneity of the plant material. For instance, the accuracy of the NIRS equation developed to quantify ergovaline on a single tall fescue cultivar (RSQ = 0.93) [27] was more accurate than the one reported by Soto-Barajas et al. [9] (RSQ = 0.76) developed on a mixed set of wild and commercial plants. Roberts et al. [43] indicated that the precision of the prediction may be affected by the concentration of the alkaloid in the plant; this could explain why the prediction models for the total amount of lolines and for NFL are more precise than the ones for NAL and NANL, which are less concentrated and account for only 10%–15% of the total amount of lolines. Moreover, when working with loline alkaloids, interferences by other lolines or their precursors cannot be excluded due to their very similar chemical structure. This could have biased the external validation of the less concentrated alkaloids decreasing the precision of the calibration and resulting in significant differences with the GC measurements. But this suggestion should be validated because herein the near infrared region was studied and not mid-infrared region, where it is easier to attribute differences of absorbance to specific chemical bounds and to identify their possible origins [27].

Unlike ergovaline or lolitrem B, loline alkaloids are not toxic to livestock, therefore although precise measurements are important, there are not legal thresholds that have to be taken into account and that cannot be overcome in commercial varieties. Loline alkaloids are correlated with activity against *Costelytra zealandica* at concentrations greater than 450 ppm [44], against the Argentine stem weevil at concentrations greater than 400 ppm [45] and against *Schizaphis graminum* at concentrations in the range of 67 to 576 ppm [46]. These toxic levels indicate that the SECV for total lolines (667 ppm) or for NFL (520 ppm) would not be inconvenient to detect plant samples with high levels of lolines (above 570 ppm) which are desirables for insect deterrence. Note that the range of total lolines concentration was 101–5629 ppm and *E. uncinata* infected plants are commonly found with lolines levels from a few thousand parts per million [47] up to 16,000 ppm and above [48]. The measurement of the exact concentration of each of the three loline alkaloids might be interesting for some studies, but in terms of insect resistance, the most studied trait is the total amount of lolines [28,49] which can deter insects from feeding on the host or have insecticide effects. Large-scale screenings are used to isolate strains producing high levels of loline alkaloids to artificially inoculate them in elite grass varieties [36]. In this respect NIRS can be used for a fast and relatively cheap preliminary screening to isolate the most performing strains, where the high levels of lolines are only slightly affected from the SECV.

As regards mycelial biomass, NIRS provides a good estimate (RSQ = 0.75; RPD = 2.04) of the endophyte concentration in the host, comparable to the one obtained by Tamburini et al. [50] which used NIRS to quantify the concentration *Fusarium proliferatum* in garlic (RSQ = 0.76; RPD = 2.04). Accuracy can be further improved by increasing the number of samples or by using more homogenous plant material with a smaller range of concentrations. This method is suitable for screening a large number of samples when manual counting of hyphae would be too laborious, and it is cheaper than using ELISA and qPCR based protocols. It would be interesting to know how NIRS can detect fungal mycelium, if through detection of structural compounds of the fungus, i.e., ergosterol a component of fungal cell membranes, or other compounds.

## 5. Conclusions

Near infrared spectroscopy is a versatile analytical technique currently used in many different fields, including grass breeding. No matter the constituents of interest, the first required step is the acquisition of the near infrared spectrum of the sample. Once the spectrum has been measured, the information that can be inferred from it are only limited by the calibrations available on the compounds of interest.

Our results showed that the use of NIRS and chemometrics allows the quantification of loline alkaloids and mycelial biomass in a heterogeneous set of meadow fescue samples. The accuracy of the equation is affected by several variables, among which there is the concentration of the analyte, therefore the prediction of the total amount of lolines and of NFL were more accurate than the ones of NANL, NAL, and with similar precision to the conventional (GC) methods. Since NIRS is already used in routine analysis of forage, alkaloid measurement can be easily implemented when testing *Epichloë*-infected varieties. This is the first report of the near-infrared spectroscopy for analysis of *Epichloë* fungal biomass in planta, a trait with important implications that has been rarely investigated in the current literature.

Although in this investigation *S. pratensis* was evaluated as the experimental plant material, this technique might be useful in similar situations for other forage species infected with *Epichloë* endophytes, providing an important parameter to scientists involved in routine forage quality research and nutritional analyses of feedstuffs.

## Figures and Tables

**Table 1:** Characteristics and number of the meadow fescue samples (*n*) used in the development of near infrared spectroscopy (NIRS) models for quantification of Loline alkaloids and mycelial mass.

Parameter	Statistical Descriptor	Training/Calibration Set	Validation Set
*N*-acetylloline (NAL)	*n*	143	46
	Range (mg∙kg^−1^)	31–320	25–339
	Mean (mg∙kg^−1^)	145	157
	SD (mg∙kg^−1^)	66	82
*N*-acetylnorloline (NANL)	*n*	142	38
	Range (mg∙kg^−1^)	25–982	43–804
	Mean (mg∙kg^−1^)	295	332
	SD (mg∙kg^−1^)	189	201
*N*-formylloline (NFL)	*n*	146	47
	Range (mg∙kg^−1^)	60–4327	77–2990
	Mean (mg∙kg^−1^)	1222	1208
	SD (mg∙kg^−1^)	767	773
Total Lolines	*n*	146	51
	Range (mg∙kg^−1^)	101–5629	107–3893
	Mean (mg∙kg^−1^)	1658	
	SD (mg∙kg^−1^)	1009	1038
Mycelial biomass	*n*	64	22
	Range (mg g^−1^)	0.220–3.960	0.360–3.970
	Mean (mg g^−1^)	1.500	1.355
	SD (mg g^−1^)	0.914	0.783

**Table 2:** Number of principal components, variability explained, and outliers detected for each of the mathematical transformations resulted after principal component analysis on the NIR spectra of the *Epichloë*-infected meadow fescue samples. The mathematical treatments are indicated as follows: *n* = no scattering; s = standard normal variate (SNV); d = correction of trend (DT); and m = application of SNV + DT transformations. The smoothing, gaps and derivatives are indicated with a number next to the letter that indicate the scatter treatment: 0 = (0,0,1,1); 1 = (1,4,4,1); 2 = (2,4,4,1), 3 = (2,10,10,1); and, 4 = (2,8,6,1).

Mathematical Treatment	Principal Components	Variability Explained (%)	Spectral Outliers
n0	9	99.99	4
n1	13	99.64	6
n2	23	99.02	2
n3	13	99.83	6
n4	15	99.68	6
s0	12	100	8
s1	15	99.55	8
s2	27	98.91	4
s3	16	99.78	8
s4	17	99.66	8
d0	10	99.95	7
d1	12	99.72	6
d2	23	99.02	2
d3	13	99.83	6
d4	15	99.68	6
m0	13	99.97	9
m1	15	99.66	8
m2	27	98.88	4
m3	16	99.78	8
m4	17	99.65	8

**Table 3:** Statistical parameters obtained from the equations developed for quantification of in planta concentrations of loline alkaloids and fungal biomass applying the modified partial least squares regressions in the NIR spectra of the meadow fescue samples.

	NAL	NANL	NFL	LOLINES	MYCELIUM
**Principal Component Analysis (PCA)**					
Pre-treatment ^†^	n3	s2	n0	n0	s0
Number of principal components (PCs)	13	27	9	9	4
Explained variability (%)	99.75	98.91	99.99	99.99	98.91
Spectral outliers (*H* > 3.0)	6	4	4	4	4
**Modified Partial Least Squares (MPLS)**					
Pre-treatment ^†^	s2	m2	m2	s2	d2
Number of samples	143	142	146	146	65
Standard deviation (SD) (mg∙kg^−1^)	65	189	767	1008	0.907
Range (mg∙kg^−1^)	31–320	25–982	60–4327	101–5629	0.22–3.96
Chemical outliers (*T* > 2.5)	6	5	7	7	2
Multiple correlation coefficient (RSQ)	0.765	0.836	0.893	0.897	0.729
Standard error of calibration (SEC) (mg∙kg^−1^)	32	77	251	324	0.473
Standard error of cross validation (SECV) (mg∙kg^−1^)	51	141	520	667	0.78
Number of PLS factors	10	10	10	10	10
Groups in cross-validation	6	6	6	6	6
**Internal Validation**					
Standard error of prediction (SEP) (mg∙kg^−1^)	31	74	240	310	0.449
Medium value of the residuals (BIAS) (mg∙kg^−1^)	0	0	0	0	−0.003
SEP corrected by the Bias (SEPc) (mg∙kg^−1^)	31	74	241	311	0.453
Multiple correlation coefficient (RSQ)	0.778	0.846	0.901	0.905	0.754
Ratio performance deviation (RPD)	2.129	2.559	3.195	3.252	2.036
**External Validation**					
Root mean standard error (RMSE = SEP) (mg∙kg^−1^)	84	184	718	894	0.979
Average residual (mg∙kg^−1^)	66	152	535	665	0.796
Student’s *t*-test (*p*)	0.018	0.048	0.265	0.157	0.894

Mycelial concentration is expressed in mg·g^−1^. ^†^ Transformation of the NIR spectra: *n* = no scattering; s = standard normal variate (SNV). The smoothing, gaps and derivatives are indicated with the a number next to the letter: 0 = (0,0,1,1); 2 = (2,4,4,1); 3 = (2,10,10,1).

## References

[1] Deaville E.R., Flinn P.C. (2000). Near-infrared (NIR) spectroscopy: An alternative approach for the estimation of forage quality and voluntary intake. Forage Evaluation in Ruminant Nutrition.

[2] Osborne B.G. (2000). Near-infrared spectroscopy in food analysis. Encyclopedia of Analytical Chemistry.

[3] Beever D.E., Mould F.L. (2000). Forage evaluation for efficient ruminant livestock production. Forage Evaluation in Ruminant Nutrition.

[4] Givens D.I., Deaville E.R. (1999). The current and future role of near infrared reflectance spectroscopy in animal nutrition: A review. Aust. J. Agric. Res..

[5] Norris K.H., Hart J.R. (1996). 4. Direct spectrophotometric determination of moisture content of grain and seeds. J. Near Infrared Spectrosc..

[6] Norris K.H., Barnes R.F., Moore J.E., Shenk J.S. (1976). Predicting forage quality by infrared replectance spectroscopy. J. Anim. Sci..

[7] Shenk J.S. (1993). Early history of forage and feed analysis by NIR 1972–1983. NIR News.

[8] Park R.S., Gordon F.J., Agnew R.E., Barnes R.J., Steen R.W.J. (1997). The use of Near Infrared Reflectance Spectroscopy on dried samples to predict biological parameters of grass silage. Anim. Feed Sci. Technol..

[9] Soto-Barajas M.C., Zabalgogeazcoa I., González-Martin I., Vázquez-de-Aldana B.R. (2017). Qualitative and quantitative analysis of endophyte alkaloids in perennial ryegrass using near-infrared spectroscopy. J. Sci. Food Agric..

[10] Barrière Y., Guillet C., Goffner D., Pichon M. (2003). Genetic variation and breeding strategies for improved cell wall digestibility in annual forage crops. A review. Anim. Res..

[11] Soto-Barajas M.C., Zabalgogeazcoa I., González-Martin I., Vázquez-de-Aldana B.R. (2018). Near-infrared spectroscopy allows detection and species identification of *Epichloë* endophytes in *Lolium perenne*. J. Sci. Food Agric..

[12] Leuchtmann A., Bacon C.W., Schardl C.L., White J.F., Tadych M. (2014). Nomenclatural realignment of *Neotyphodium* species with genus *Epichloë*. Mycologia.

[13] Schardl C.L. (1996). *Epichloë* species: Fungal symbionts of grasses. Annu. Rev. Phytopathol..

[14] Johnson L.J., De Bonth A.C.M., Briggs L.R., Caradus J.R., Finch S.C., Fleetwood D.J., Fletcher L.R., Hume D.E., Johnson R.D., Popay A.J. (2013). The exploitation of *Epichloë* endophytes for agricultural benefit. Fungal Divers..

[15] Saikkonen K., Young C.A., Helander M., Schardl C.L. (2016). Endophytic *Epichloë* species and their grass hosts: From evolution to applications. Plant Mol. Biol..

[16] Panaccione D.G., Beaulieu W.T., Cook D. (2014). Bioactive alkaloids in vertically transmitted fungal endophytes. Funct. Ecol..

[17] Clay K., Schardl C. (2002). Evolutionary origins and ecological consequences of endophyte symbiosis with grasses. Am. Nat..

[18] Porter J.K., Thompson F.N. (1992). Effects of fescue toxicosis on reproduction in livestock. J. Anim. Sci..

[19] Gallagher R.T., White E.P., Mortimer P.H. (1981). Ryegrass staggers: Isolation of potent neurotoxins lolitrem A and lolitrem B from staggers-producing pastures. N. Z. Vet. J..

[20] Leuchtmann A., Schmidt D., Bush L.P. (2000). Different levels of protective alkaloids in grasses with stroma-forming and seed-transmitted *Epichloë/Neotyphodium* endophytes. J. Chem. Ecol..

[21] Popay A.J., Tapper B.A., Podmore C. (2009). Endophyte-infected meadow fescue and loline alkaloids affect argentine stem weevil larvae. N. Z. Plant Prot..

[22] Nelli M.R., Scheerer J.R. (2016). Synthesis of peramine, an anti-insect defensive alkaloid produced by endophytic fungi of cool season grasses. J. Nat. Prod..

[23] Lee S.T., Gardner D.R., Cook D. (2017). Identification of indole diterpenes in *Ipomoea asarifolia* and *Ipomoea muelleri*, plants tremorgenic to livestock. J. Agric. Food Chem..

[24] Panaccione D.G., Ryan K.L., Schardl C.L., Florea S. (2012). Analysis and modification of ergot alkaloid profiles in fungi. Methods in Enzymology.

[25] Baldauf M.W., Mace W.J., Richmond D.S. (2011). Endophyte-mediated resistance to black cutworm as a function of plant cultivar and endophyte strain in tall fescue. Environ. Entomol..

[26] Roberts C.A., Joost R.E., Rottinghaus G.E. (1997). Quantification of ergovaline in tall fescue by near infrared reflectance spectroscopy. Crop Sci..

[27] Pan J., Bhardwaj M., Nagabhyru P., Grossman R.B., Schardl C.L. (2014). Enzymes from fungal and plant origin required for chemical diversification of insecticidal loline alkaloids in grass-*Epichloë* symbiota. PLoS ONE.

[28] Patchett B.J. (2007). Loline alkaloids: Analysis and Effects on Sheep and Pasture Insects. Ph.D. Thesis.

[29] Schardl C.L., Grossman R.B., Nagabhyru P., Faulkner J.R., Mallik U.P. (2007). Loline alkaloids: Currencies of mutualism. Phytochemistry.

[30] Freitas P. (2017). Crossing the Species Barrier: Investigating Vertical Transmission of a Fungal Endophyte from Tall Fescue within a Novel Ryegrass Association. Ph.D. Thesis.

[31] Spiering M.J., Greer D.H., Schmid J. (2006). Effects of the fungal endophyte, *Neotyphodium lolii*, on net photosynthesis and growth rates of perennial ryegrass (*Lolium perenne*) are independent of in planta endophyte concentration. Ann. Bot..

[32] Panaccione D.G., Johnson R.D., Wang J., Young C.A., Damrongkool P., Scott B., Schardl C.L. (2001). Elimination of ergovaline from a grass-*Neotyphodium* endophyte symbiosis by genetic modification of the endophyte. Proc. Natl. Acad. Sci. USA.

[33] Rasmussen S., Parsons A.J., Bassett S., Christensen M.J., Hume D.E., Johnson L.J., Johnson R.D., Simpson W.R., Stacke C., Voisey C.R. (2007). High nitrogen supply and carbohydrate content reduce fungal endophyte and alkaloid concentration in *Lolium perenne*. New Phytol..

[34] Young C.A., Bryant M.K., Christensen M.J., Tapper B.A., Bryan G.T., Scott B. (2005). Molecular cloning and genetic analysis of a symbiosis-expressed gene cluster for lolitrem biosynthesis from a mutualistic endophyte of perennial ryegrass. Mol. Genet. Genom..

[35] Faville M.J., Briggs L., Cao M., Koulman A., Jahufer M.Z.Z., Koolaard J., Hume D.E. (2015). A QTL analysis of host plant effects on fungal endophyte biomass and alkaloid expression in perennial ryegrass. Mol. Breed..

[36] Cagnano G., Roulund N., Jensen C.S., Forte F.P., Asp T., Leuchtmann A. (2019). Large scale screening of *Epichloë* endophytes infecting *Schedonorus pratensis* and other forage grasses reveals a relation between microsatellite-based haplotypes and loline alkaloid levels. Front. Plant Sci..

[37] Hiatt E.E., Hill N.S., Bouton J.H., Stuedemann J.A. (1999). Tall fescue endophyte detection: Commercial immunoblot test kit compared with microscopic analysis. Crop Sci..

[38] Cagnano G., Lenk I., Roulund N., Jensen C.S., Cox M.P., Asp T. (2020). Mycelial biomass and concentration of loline alkaloids driven by complex population structure in *Epichloë uncinata* and meadow fescue (*Schedonorus pratensis*). Mycologia.

[39] Barnes R.J., Dhanoa M.S., Lister S.J. (1989). Standard normal variate transformation and de-trending of near-infrared diffuse reflectance spectra. Appl. Spectrosc..

[40] Naes T., Isakson T., Fearn T., Davies T. (2002). A User-Friendly Guide to Multivariate Calibration and Classification.

[41] Shenk J.S., Westerhaus M. (1995). Routine Operation, Calibration, Development and Networksystem Management Manual.

[42] Martens H., Martens M. (2001). Multivariate analysis of quality. An introduction. Meas. Sci. Technol..

[43] Williams P., Sobering D., Davies A.M.C., Williams P.C. (1996). How do we do it: A brief summary of the methods we use in developing near infrared calibrations. Near Infrared Spectroscopy: The Future Waves.

[44] Patchett B., Gooneratne R., Chapman B., Fletcher L. (2011). Effects of loline-producing endophyte-infected meadow fescue ecotypes on New Zealand grass grub (*Costelytra zealandica*). N. Z. J. Agric. Res..

[45] Patchett B.J., Chapman R.B., Fletcher L.R., Gooneratne S.R. (2008). Endophyte infected *Festuca pratensis* containing loline alkaloids deters feeding by *Listronotus bonariensis*. N. Z. Plant Prot..

[46] Wilkinson H.H., Siegel M.R., Blankenship J.D., Mallory A.C., Bush L.P., Schardl C.L. (2000). Contribution of Fungal Loline Alkaloids to Protection from Aphids in a Grass-Endophyte Mutualism. Mol. Plant Microbe Interact..

[47] Patchett B., Gooneratne R., Fletcher L., Chapman B. (2009). Seasonal changes in leaf and stem loline alkaloids in meadow fescue. Crop Pasture Sci..

[48] Barker G.M.G., Patchett B.J., Cameron N.E.N. (2015). *Epichloë uncinata* infection and loline content afford Festulolium grasses protection from black beetle (*Heteronychus arator*). N. Z. J. Agric. Res..

[49] Levasseur C., Pinson-Gadais L., Kleiber D., Surel O. (2010). Near infrared spectroscopy used as a support to the diagnostic of *Fusarium* species. Rev. Med. Vet..

[50] Tamburini E., Mamolini E., De Bastiani M., Marchetti M.G. (2016). Quantitative determination of *Fusarium proliferatum* concentration in intact garlic cloves using near-infrared spectroscopy. Sensors.

